# Need for informed providers: exploring LA-PrEP access in focus groups with PrEP-indicated communities in Baltimore, Maryland

**DOI:** 10.1186/s12889-024-18595-7

**Published:** 2024-05-08

**Authors:** Rose Pollard Kaptchuk, Amber M. Thomas, Amit “Mickey” Dhir, Sunil S. Solomon, Steven J. Clipman

**Affiliations:** 1 Johns Hopkins Bloomberg School of Public Health, Baltimore, MD USA; 2 Johns Hopkins University School of Nursing, Baltimore, MD USA; 3 Johns Hopkins University School of Medicine, Baltimore, MD USA

**Keywords:** Long-acting PrEP, Sexual and gender minorities, Key populations, HIV prevention, Provider Education, Accessibility

## Abstract

**Background:**

The approval of long-acting pre-exposure prophylaxis PrEP (LA-PrEP) in the United States brings opportunities to overcome barriers of oral PrEP, particularly among sexual and gender minority communities who bear a higher HIV burden. Little is known about real-time decision-making among potential PrEP users of LA-PrEP post-licensure.

**Methods:**

We held focus group discussions with people assigned male at birth who have sex with men in Baltimore, Maryland to explore decision-making, values, and priorities surrounding PrEP usage. A sexual and gender minority-affirming health center that provides PrEP services supported recruitment. Discussions included a pile-sorting activity and were audio-recorded. Recordings were transcribed and analyzed iteratively, combining an inductive and deductive approach.

**Results:**

We held five focus groups from Jan-June 2023 with 23 participants (21 cisgender men who have sex with men, two transgender women who have sex with men; mean age 37). Among participants, 21 were on oral PrEP, one was on injectable PrEP, and one had never taken PrEP. Most had never heard about LA-PrEP. When making decisions about PrEP, participants particularly valued efficacy in preventing HIV, side effects, feeling a sense of security, and ease of use. Perceptions varied between whether oral or injectable PrEP was more convenient, but participants valued the new opportunity for a choice in modality. Factors influencing PrEP access included cost, individual awareness, provider awareness, and level of comfort in a healthcare environment. Participants emphasized how few providers are informed about PrEP, placing the burden of being informed about PrEP on them. Comfort and trust in a provider superseded proximity as considerations for if and where to access PrEP.

**Conclusions:**

There is still low awareness about LA-PrEP among sexual and gender minority communities; thus, healthcare providers have a critical role in influencing access to LA-PrEP. Despite this, providers are still vastly underinformed about PrEP and underprepared to support clients in contextualized ways. Clients are more likely to engage in care with affirming providers who offer non-judgmental conversations about sex and life experiences. Provider education in the United States is urgently needed to better support clients in choosing a PrEP modality that is right for them and supporting adherence for effective HIV prevention.

## Background

The HIV epidemic in the United States (U.S.) continues to pose significant public health challenges, yet this burden is not distributed equally. Of the 36,136 estimated new HIV diagnoses in 2021, 52% were in the Southern U.S., 71% were among men who have sex with men, and 2% were among transgender people [1, 2]. Entrenched social determinants of health drive rates of new HIV diagnoses, with significant racial and ethnic disparities [3–5]. HIV prevention tools that could reduce inequities are expanding. In 2012, the U.S. Food and Drug Administration (FDA) approved the use of oral pre-exposure prophylaxis (PrEP), a medicine that reduces the chances of getting HIV from sex by 99% when taken as prescribed [6, 7]. The disparities in new HIV diagnoses permeate through PrEP access and usage in the U.S. In 2022, only 36% of people who could benefit from PrEP were prescribed it [8]. This gap is particularly high in Baltimore, Maryland [9]. In Baltimore, 61% of new HIV diagnoses in 2022 were among men who have sex with men [10], yet a 2021 study found that only 19% of PrEP-indicated men who have sex with men in Baltimore were prescribed PrEP, with disparities among non-Hispanic Black populations [11]. 

In 2021, the FDA approved a long-acting version of PrEP (LA-PrEP) [12]. Instead of PrEP only being available as a pill, LA-PrEP introduced an injectable option administered by a trained provider, first as two injections one month apart, followed by an injection every two months. The advancement of LA-PrEP can make significant progress toward ending the HIV epidemic in the U.S. compared to oral PrEP alone [13, 14]. LA-PrEP brings opportunities to overcome barriers of oral PrEP related to convenience and privacy [15–17]. However, most of what we know about the acceptability of LA-PrEP is from studies conducted pre-licensure [16, 18–21]. A 2020 literature review of barriers to PrEP in the United States across quantitative and qualitative studies highlighted awareness, stigma, cost, and distrust of healthcare systems as key barriers prior to LA-PrEP rollout [22]. Potential facilitators for PrEP accessibility offered by LA-PrEP documented in recent literature include periodic (instead of daily) administration which may reduce logistical barriers and social stigma [23]. However, since FDA approval of LA-PrEP, access has been hampered by challenges in the logistics of service provision and user insurance coverage, [24–26] as well as low awareness and low trust in LA-PrEP [27]. While estimates are not widely available, ViiV Healthcare reported that 11,000 people were taking LA-PrEP in the U.S. as of 2023 [28]. To improve uptake of LA-PrEP, a better understanding of acceptance of LA-PrEP post-licensure and real-time decision-making among potential PrEP users is needed, as well as how healthcare programs can support individual-level choice [13, 15, 16]. As PrEP options continue to expand and evolve, this research is critical to close the gap between PrEP access barriers and PrEP uptake, especially among higher-HIV-burden communities in the U.S.

Our study presents qualitative insight into PrEP decision-making among PrEP-indicated communities in Baltimore, Maryland. The goal of this study was to explore decision-making, values, and priorities surrounding PrEP usage among people assigned male at birth who have sex with men in Baltimore, Maryland, given the newly available injectable LA-PrEP option. Understanding why individuals may or may not choose LA-PrEP over oral forms can inform strategies to improve PrEP uptake and adherence and can help tailor support mechanisms to people’s specific needs.

## Methods

### Theoretical grounding

Our study is grounded in the multidimensional model of healthcare access proposed by Penchansky and Thomas [29]. This framework extends the concept of access beyond individual characteristics, emphasizing the synergy between individual and organizational factors. It delineates the construct of access into five core dimensions: availability, accessibility, affordability, accommodation, and acceptability. Saurman proposed a sixth dimension, awareness, which addresses communication and information strategies on clients’ understanding and health literacy [30]. This framework embraces a comprehensive view of the healthcare environment and services, which aligns with our research team’s consideration of organizational factors as impactful in shaping access to care.

### Study overview

To explore factors influencing PrEP decision-making, access to care, and retention in care, we facilitated focus group discussions among PrEP-indicated individuals in Baltimore, Maryland, from January to June 2023. Focus groups were designed to explore collective and individual attitudes, beliefs, and behaviors regarding PrEP at the community level [31, 32]. The focus groups were the first phase of a mixed methods study — they were also designed to inform a second phase comprised of an online survey. Given the substantial HIV burden among sexual minority men in the U.S., we designed our study to target cisgender men who have sex with men; however, we did not exclude transgender women from participating who met our eligibility criteria (i.e., assigned male at birth and self-report of sex with a man in the last 12 months). Our research was carried out in collaboration with a sexual and gender minority-affirming health center that provides PrEP services in the region.

### Recruitment

Healthcare providers and staff at the sexual and gender minority-friendly healthcare center supporting this work informed potential participants about our study and distributed informational posters that included a contact number for those interested in participating. Additionally, we expanded our recruitment efforts through social media campaigns and posters at various sexual and gender minority-friendly venues across Baltimore. Potential participants initiated contact by calling the provided number which linked them to our research team. During this initial call, we conducted pre-screening to confirm eligibility, arranged focus group appointments, and obtained verbal consent. Eligible focus group participants were assigned male at birth, 18 years or above, living in the Baltimore metropolitan area, self-reported oral or anal sex with a man in the past 12 months, and self-reported a negative or unknown HIV status.

### Data collection

Focus group discussions took place in a private conference room either at the sexual and gender minority-friendly healthcare center or at the Johns Hopkins University campus. Focus groups were facilitated by one moderator, with other study team members present to take notes and supplement questions. The moderator used a semi-structured focus group guide, which covered perceptions of and experiences with different PrEP modalities, norms, and communication surrounding PrEP, experiences with healthcare, and the impact of PrEP on sex behaviors. Focus groups began with a pile sorting activity to elicit factors that participants perceived as the most impactful in making decisions about PrEP [33–35]. To preserve the self-defined wording of PrEP decision-making factors during the pile sorting activity, we did not offer example factors prior to participants sharing what they wrote down on index cards. Discussions were audio recorded. Each participant received $50 USD in cash at the end of the focus group.

### Data analysis

We recorded what participants wrote on note cards during the pile sorting activity into an Excel spreadsheet and discussed variation in content and terminology across the focus groups. We transcribed audio recordings using Otter.ai, a speech-to-text application. Author AMT listened to each recording and refined transcripts for accuracy. AMT and RPK created an initial codebook by independently coding the same transcript and then discussing and refining the codebook. We employed *Process Coding* to track the temporal sequence of events or experiences, *Values Coding* to understand participants’ priorities, beliefs, and viewpoints, and *Emotions Coding* to capture the affective responses of participants [36]. We inductively generated sub-codes and additional categories based on emergent data patterns. AMT applied these codes to all transcripts using ATLAS.ti software, inductively adding new codes as relevant. Regular team discussions facilitated the integration of new codes and resolved ambiguities. AMT and RPK then reviewed the coded data and drafted analytical memos to capture salient points per code. These memos were collectively examined to distill overarching themes and to document how themes varied across participant demographics.

### Positionality

Researchers on our study team have diverse racial and sexual identities, and all have experience with communities affected by HIV through lived experience, research, or clinical service provision. Focus groups were primarily led by a cisgender, White woman with expertise in qualitative methodology. Study methodology, data collection, and data analysis were shaped by collaborative communication across the study team to synthesize our experience and understanding of PrEP and norms among queer communities in Baltimore.

## Results

A total of 23 participants attended one of five focus group discussions between January and June 2023. Group size per focus group ranged from three to six. Participant ages ranged from 22 to 75 years (mean 37); 21 were men who have sex with men, and two were transgender women who have sex with men; 12 identified as White, six as Black, three as Asian, and two as Hispanic. Among the total participants, 21 were on oral PrEP at the time of the focus group, one was on injectable PrEP, and one had never taken PrEP. Most had never heard about LA-PrEP before. Each focus group lasted between one and a half to two hours. Across focus groups, participants discussed their values related to PrEP and how these values related to priorities for PrEP access. We first present salient values about PrEP which participants discussed and then explore priorities of PrEP accessibility.

### Values about PrEP

#### Efficacy

A recurring theme in participants’ considerations of PrEP usage was their perception of the medication’s effectiveness in preventing HIV infection.

*“If I found out that a bunch of people that were on PrEP had like, gotten HIV, like it wasn’t really working, I would probably stop taking it.*” (Man, age 27, White)

Participants acknowledged gaps in their knowledge about PrEP options, expressing a keen interest in learning more about the efficacy of different PrEP modalities, including those currently under investigation. The degree to which PrEP is effective was frequently highlighted as a critical factor, with some individuals characterizing it as “the most impactful thing” in their decision-making.

#### Bodily impact

The potential side effects and overall bodily impact of PrEP were significant factors for participants when considering its use. Participants shared accounts of adverse experiences with oral PrEP, such as discomfort and severe nausea. One Black transgender woman, age 35, shared how side effects led her to discontinue PrEP:

*"I had a whole trash bag for the throw up, and it was just lying on my stomach. I just cut [PrEP] off completely."*

The severity of side effects influenced participants’ perceptions of PrEP’s safety. Additionally, there was concern about the long-term bodily effects of PrEP, particularly among those with pre-existing health conditions or those taking other medications.

#### Convenience

Participants emphasized the value of convenience and ease of use of PrEP, whether it was oral or injectable PrEP.

*“You have so many things going on in your life so it’s like really hard to track [taking PrEP]…you have to make a habit of like doing that. And it’s like, injecting yourself [every two months] would ease that…it’s the convenience part.”* (Man, age 33, Asian)

Some participants, particularly older individuals and those already accustomed to a daily medication regimen, reported no inconvenience with daily oral PrEP. In contrast, others highlighted the challenge of daily adherence and the anxiety associated with potentially missing a dose. A participant (Man, age 33, Hispanic) mentioned physical discomfort in swallowing pills.

The discussions around injectable PrEP revealed mixed feelings. The advantage of less frequent dosing was appealing to some. A participant (Man, age 45, Black) described relief from the daily responsibility and associated anxiety:

Conversely, others elevated the inconvenience of having to deal with more frequent clinic visits and the discomfort of having an injection:

*“I thought about the injectable version that’s now been approved, but I have a terrible phobia for needles. So it’s like, do I want that? No.”* (Man, age 22, White)

An overarching narrative was the desire for PrEP to seamlessly integrate into an individual’s lifestyle with minimal disruption. Participants appreciated having a choice between modalities (oral vs. injectable) and dosing strategies (daily vs. event-driven), emphasizing the importance of personalized PrEP options. During the pile sorting activity, a participant likened the preference for convenience to choosing the simplest method for a task.

*“It goes back to one more I put down, which is ease– like it’s easily accessible. Would you rather cook a pizza outside on a really hot sunny day or put it in a microwave? Like which one will be easier for you?… I’d rather work smarter, not harder. ”* (Man, age 33, Asian)

#### Sense of security

A sense of security from PrEP was another recurring theme among participants, who associated its use with peace of mind and an enhanced feeling of control over their health. Participants particularly valued regular PrEP care, which provided face-to-face touchpoints with a healthcare provider and brought reassurance about HIV prevention and overall health.

“*The STI testing every three months is a really comforting thing… there’s a comforting level both for myself and for partners that every three months at least I know I’ve gone in, I’ve done the best that I can to try and not have anything transmitted.*” (Man, age 63, White)

Participants voiced that if it were not for being on PrEP, they likely would not get sexually transmitted infection (STI) tests done as frequently.

### Priorities for PrEP accessibility

#### Paying for PrEP

The financial aspect of accessing PrEP was a central topic in the focus groups, with participants identifying cost as “the obvious” barrier to accessibility. Discussions about cost were intertwined with discussions about insurance. Participants’ confidence in their insurance plans to cover PrEP significantly influenced their decisions to access the medication, especially when considering LA-PrEP. A Black man participant, age 52, shared how insurance limitations can even compel people to ration their PrEP intake:

*I had lost my job, and I didn’t have any more insurance and went out to save my PrEP. I would take it every other day until I got another job and got some insurance so I can go back to getting it all the time.*

In order to access PrEP, participants emphasized the importance of understanding how to navigate insurance complexities, which impacted their preferences about where to go to get PrEP. Participants prioritized clinics where they felt confident their insurance would be accepted to cover the direct and associated costs of PrEP. Often, these components surrounding cost superseded proximity in choosing a location to access PrEP.

#### Personal awareness about PrEP

Outside of payment options, awareness of PrEP emerged as a crucial factor in its accessibility, with participants reflecting on how they initially learned about it. Many first heard about PrEP through word of mouth within their social circles or by observing friends or acquaintances who were PrEP users. Digital platforms, including social media, online advertisements, and various internet resources, also played a significant role in raising awareness, particularly for those from families or communities with limited acceptance of sexual and gender minorities.

*“It should become more commonplace if it took me five years to even hear about [PrEP] through a friend. Like how many other people didn’t know about it?” (Man, age 30, White)*

Participants shared how conversations about PrEP are commonplace among gay communities, which results in widespread awareness about PrEP among certain groups of friends or networks. This often felt in contrast to interactions with heterosexual communities or the broader society in which participants live, where conversations about PrEP are few and far between.

*“Even my straight friends…if they happen to like, ask me about [PrEP] and I explain to them what it is, they are just like, astonished that that’s even a real thing.” (Man, age 27, White)*

Participants pointed out that the lack of awareness about PrEP in heterosexual communities could restrict broader population access to PrEP. Additionally, the responsibility to inform and educate often falls on the shoulders of queer community members, which can be burdensome and complex, especially for those dealing with stigma or not openly gay. An Asian man participant, age 34, shared:

#### Provider awareness about PrEP

Participants voiced an expectation that healthcare providers should help promote awareness about PrEP but that their experiences often stood in stark contrast. Participants repeatedly described encounters with providers who “had absolutely no idea” about PrEP, were “not comfortable prescribing it,” or even encouraged them to stop taking PrEP. For example, one White man participant, age 35, shared his experience with a provider:

“*He asked what my HIV numbers were. And I was like, oh, it’s preventative, it’s PrEP. And he was like, no, it’s not, I know what it is. And I was like, uhh, I know you’re a doctor, and you’re smarter than me in every way, but you are not right about this. And we like went back and forth. I was very uncomfortable*.”

Other participants resonated with this example shared in the focus group, particularly how awkward, embarrassing, and draining it feels to navigate power dynamics with a provider whose PrEP awareness falls short of expectations.

Experience with “medical professionals who don’t fully get it” was particularly apt when discussing newer modalities of PrEP. One participant (Man, age 22, White) shared an experience of asking a provider when they would start offering injectable PrEP, and the provider's response was, *“Oh, I didn’t even know that was a thing.”* There was a sense among participants that they were learning about PrEP advancements at the same time as providers. This often placed participants in the role of being more knowledgeable about PrEP than their providers. While for many this felt like a burden, one participant shared how this contributed to a feeling of cooperation: *“I feel like we’re learning all together, especially with the injectable.” (Transgender woman, age 32, Black).*

Participants expressed a desire for healthcare providers to be well-informed about PrEP, including different modalities, effects on the body, and management of potential side effects. They saw providers as having a significant opportunity to educate clients about PrEP, particularly for those who lack supportive communities for open discussion. The focus groups conveyed a collective disappointment with healthcare providers who did not meet these expectations, emphasizing the need for improved provider education and engagement with PrEP as a public health intervention.

#### Building trust with providers

Beyond feeling confident in a provider’s knowledge about PrEP, establishing trust with a provider was another important element of PrEP accessibility. Participants emphasized that a PrEP provider should be comfortable talking about sex openly, without judgment. They should have an authentic investment in what a client says about sex and relationships rather than mechanically asking “check the boxes” behavioral questions. They also wanted a PrEP provider with whom they could openly discuss the experience of being queer in a heteronormative society. One participant shared how big of a difference this can make:

*“Just knowing that [a healthcare provider] is a safe space to come to is like a big thing because, like [another participant] said, not everybody is as knowledgeable or like, kind of understands just some of the concerns that you might have as you know, a queer man, and so I think that provides a level of comfort.” (Man, age 29, White)*

Participants felt that when a person feels open and heard, they are more likely to come back to a PrEP provider. If not, people may shield information or not attend clinic visits. Feeling comfortable with a provider went beyond talking about sexual experiences to a provider engaging with and affirming their full identity. For a Black transgender woman participant, age 32, this was make or break:

*"I had a provider, and I was like, I don’t really feel like going back because I don’t feel like teaching you. If you’re not going to do the work, then I’m not even going to…if you’re not going to even address those things, and how I learned about how I’m affected and how I show up in the world, then, why would I even come back?"*

These aspects were part of the process to establish a trusting relationship with a provider, which comes with time. How much participants trusted providers and how comfortable they felt in a healthcare environment determined if, how, and where they went to access PrEP.

## Discussion

Participants in our focus groups discussed a range of values regarding PrEP decision-making, including efficacy to prevent HIV, side effects and bodily impact, convenience, and a sense of security. We identified themes of participant priorities for accessing PrEP across cost, personal and provider awareness, and comfort with providers. Our findings offer evidence in line with Penchansky and Thomas’s dimensions of healthcare access, particularly the dimensions of *affordability, acceptability*, and *awareness* as influential in PrEP access. Our study adds practicality to previous studies surrounding LA-PrEP acceptability conducted pre-licensure, as participants reflected on LA-PrEP as an option actively available to them compared with oral PrEP. Findings support studies in the U.S. demonstrating that anticipated stigma from providers, perceived benefit, and cost influence PrEP access and use [37–39]. 

Participants questioned how effective various PrEP modalities are in preventing HIV, which resonates with studies emphasizing the need to provide users with comprehensive information on PrEP effectiveness across different modalities [40, 41]. The bodily impact considerations among participants mirror previous research on the impact of side effects and perceived long-term effects on individuals’ decisions to initiate or continue PrEP [40, 42–44]. Similar to a 2019 qualitative study with men who have sex with men in Baltimore, our results demonstrate how using any mode of PrEP can bring peace of mind about having sex and preserving one’s health [45]. This extends beyond HIV prevention to a feeling of agency in taking care of overall health, aided by routine checkups with a provider [45]. This sense of security about sexual and overall health can be integrated into provider and program messaging when considering PrEP’s benefits with potential users.

Our findings demonstrate how interest in LA-PrEP among PrEP-indicated people will likely come with reservations, questions, and the need for additional information [46]. Given how long oral PrEP has been available, sexual and gender minorityindividuals may be more likely to encounter information about it in everyday life; however, this is not yet true of LA-PrEP. Many potential LA-PrEP users will likely turn to their providers to get information. Health providers across specialties offer an opportunity to provide this information and a path for shared PrEP decision-making between injectable and oral options [46]. However, our focus groups confirmed that provider ignorance about PrEP is still a major issue, reinforcing the same challenge from the past decade: the majority of healthcare providers in the U.S. lack knowledge regarding PrEP and feel uncomfortable prescribing it [47–51]. Further, providers who are informed about PrEP do not often see HIV-negative clients who may benefit most [40]. Our study verifies previous findings that clients themselves often initiate PrEP conversations with providers, which can feel like a burden, underscoring the importance for providers to be prepared for these discussions [52, 53]. 

For PrEP uptake to improve, especially among men who have sex with men, transgender people, and other communities facing higher HIV burdens, providers need to be vastly better informed and prepared to support client-specific needs. Provider education should include comprehensive information about efficacy, potential side effects, and long-term impacts across the PrEP modalities currently available [54]. In addition to PrEP-specific information, provider education should also include comprehensive training on sexual health to understand the diversity of sexual preferences, communicate without judgment about sexual behaviors, discuss sources of potential HIV/STI exposure, and support choices for HIV/STI prevention [55]. This training should extend beyond infectious disease specialists to primary care providers because they may be more likely to have first-time conversations with those indicated for PrEP [56–58]. 

Our study emphasizes the importance of building trust between individuals and their healthcare providers to promote PrEP access, which builds on research documenting the role of client-provider relationships in PrEP persistence and retention in care [59–62]. Providers need to be prepared to address anticipated stigma, medical mistrust, and perceived racism in order for potential PrEP users to trust them as partners in PrEP decision-making [43, 63, 64]. This trust is rooted in providers’ ability to create a safe, non-judgmental space and to be ready to interact with how clients show up in the world. This is especially important for people with marginalized racial identities and sexual and gender minority individuals [65–67]. Research has shown how individuals with intersecting marginalized identities, such as Black/African American and Hispanic/Latino sexual and gender minorities, have unique challenges in accessing and adhering to PrEP [68–70]. Incorporating anti-racism and sexual and gender minority competency training for staff and providers in healthcare settings is essential to ensure that all individuals can access PrEP in environments where they feel welcomed and supported [71]. 

Our results further highlight how accessibility means different things to different people, and individuals may prioritize different values about PrEP compared to others. This is in line with previous work highlighting the benefit of user choice and calling for differentiated service delivery approaches to ensure efficient and equitable PrEP access as options in the HIV prevention toolbox expand [26, 72]. The array of values in our results emphasizes the importance for providers to individualize care by tailoring conversations to what each unique person cares about. Clear communication should involve a collaborative discussion that considers individual preferences, needs, and potential challenges. Discussions can include why clients are interested in PrEP, what they are concerned about regarding PrEP, what they are excited about related to PrEP, and what their goals are with PrEP. These questions can draw out the specific values and priorities that are important to each client to aid in choosing a modality that is right for their lifestyle and goals.

Our study was limited in various ways. Most of our focus group participants were recruited from clients receiving care at the PrEP service provider in Baltimore instead of through the physical posters and social media posts. Our findings are more representative of PrEP-indicated people who are engaged in care and likely have had positive experiences with healthcare rather than those who are not active in healthcare or those with negative or traumatic experiences. Another limitation was not recruiting transgender women as a separate group for focus groups, as transgender women have unique needs and experiences from cisgender men. More work is needed to consider perceptions and accessibility of PrEP advancements among transgender women separately from cisgender men. The participatory pile sorting activity in focus groups strengthened our study. This methodology allowed us to determine which factors are most important to participants when considering and adhering to a PrEP option. We used this data to subsequently design a discrete choice experiment, which will be used in an upcoming quantitative survey focused on community and network dynamics informed by these focus groups.

Our findings underscore how values and priorities differ per person when considering LA-PrEP compared to oral PrEP. While proximity to PrEP providers is important, comfort and trust in the healthcare environment can outweigh geographical convenience. Providers are critical channels of information promoting PrEP access and should take responsibility for engaging clients in non-judgmental conversations about sex and PrEP decision-making. There is a critical need for comprehensive PrEP provider education to overcome provider-level barriers of shared decision-making so that providers can help clients choose and adhere to the PrEP modality that is right for them, thereby realizing the potential of LA-PrEP in ending the HIV epidemic in the U.S.

## Data Availability

Deidentified data generated and analyzed during this study are available from the corresponding author upon reasonable request.

## Abbreviations

FDA: U.S. Food and Drug Administration
LA-PrEP: Long-acting pre-exposure prophylaxis
PrEP: Pre-exposure prophylaxis
U.S.: United States
